# Multiomics Analysis Reveals Therapeutic Targets for Chronic Kidney Disease With Sarcopenia

**DOI:** 10.1002/jcsm.13696

**Published:** 2025-02-06

**Authors:** Meiqiu Wang, Lianghui You, Xu He, Yingchao Peng, Ren Wang, Zhiqiang Zhang, Jiaping Shu, Pei Zhang, Xiaoyi Sun, LiLi Jia, Zhengkun Xia, Chenbo Ji, Chunlin Gao

**Affiliations:** ^1^ Department of Pediatrics, Nanjing Jinling Hospital, Affiliated Hospital of Medical School Nanjing University Nanjing China; ^2^ State Key Laboratory of Reproductive Medicine and Offspring Health, Nanjing Women and Children's Healthcare Institute Women's Hospital of Nanjing Medical University, Nanjing Maternity and Child Health Care Hospital Nanjing China; ^3^ Department of Pediatrics, Nanjing Drum Tower Hospital, Affiliated Hospital of Medical School Nanjing University Nanjing China; ^4^ Department of Pediatrics Jinling Hospital, Nanjing Medical University Nanjing China; ^5^ Department of Pediatrics Medical School of Southeast University Nanjing China; ^6^ Department of Pediatrics Jinling Hospital Nanjing China

**Keywords:** chronic kidney disease, multiomics analysis, myotube, sarcopenia, skeletal muscle, Spp1

## Abstract

**Background:**

The presence of sarcopenia in patients with chronic kidney disease (CKD) is associated with poor prognosis. The mechanism underlying CKD‐induced muscle wasting has not yet been fully explored. This study investigates the influence of renal secretions on muscles using multiomics sequencing.

**Methods:**

The kidney transcriptome analysis by RNA‐seq and protein profiling by tandem mass tag (TMT), serum TMT and muscle TMT were performed in CKD established using 0.2% adenine and control mice. Spp1 recombinant protein was used to study its effect on myotube atrophy in vitro. In animal experiments on CKD, pharmacological inhibition of Spp1 was used to explore the role of Spp1 in skeletal muscle wasting. Transcriptome analysis was performed to identify differentially expressed genes (DEGs) in the gastrocnemius muscle following Spp1 pharmacological inhibition.

**Results:**

In the renal transcriptome and TMT, 503 and 377 proteins/genes respectively were co‐upregulated and co‐downregulated. In the serum TMT of CKD and normal control (NC) mice, 22 upregulated and 7 downregulated differentially expressed proteins (DEPs) showed the same expression patterns as those in the kidney transcriptome and TMT analysis. Based on bioinformatics analysis and reported studies, we selected Spp1 for further validation. Spp1 recombinant protein was added to C2C12 myotubes in vitro, and the results indicated that Spp1 significantly increased the protein levels of the muscle atrophy marker (Murf‐1) and promoted the smaller myotubes (all *p* < 0.05). Compared with NC mice, Spp1 mRNA and protein levels were significantly upregulated in the kidneys of CKD mice, and the serum concentration of Spp1 was also markedly increased (all *p* < 0.05). In animal experiments, pharmacological inhibition of Spp1 increased the weights of gastrocnemius and tibialis anterior muscles (*p* < 0.05) and improved muscle atrophy phenotype. Transcriptome analysis showed that DEGs in the gastrocnemius muscle following Spp1 pharmacological inhibition were enriched in protein digestion and absorption, glucagon signalling pathway, apelin signalling pathway and ECM‐receptor interaction pathway.

**Conclusions:**

Our study is the first to establish a regulatory network of kidney‐muscle crosstalk to explore the potential mechanism of CKD‐related sarcopenia. Employing multiomics analysis, cellular assessment and animal experiments, we have identified that Spp1 could potentialy serve as a promising therapeutic target for CKD patients with sarcopenia.

## 1 Introduction

Chronic kidney disease (CKD) is a global public health problem, affecting 10%–15% of adults worldwide [1]. By 2017, the number of patients globally had reached 697.5 million, including 132.3 million in China [2]. Sarcopenia, a wasting syndrome in which skeletal muscle strength, mass and physiological function decrease, is common in patients with CKD, especially those with end‐stage renal disease and those undergoing haemodialysis [3]. The insidious progression of sarcopenia decreases the quality of life in patients with CKD and increases falls and fractures, hospitalization rates and mortality [4].

To explore the mechanism of CKD‐induced sarcopenia, current studies have focused on molecular alterations in skeletal muscles. Mitochondrial dysfunction [5, 6], elevated oxidative stress [6, 7] and increased inflammation [8] are frequently observed in animal and human CKD muscle models. The pathogenesis of sarcopenia is complex, and it cannot be studied in isolation. The kidney secretes circulating factors that lead to skeletal muscle loss as the primary lesion and the main endocrine organ [9]. Activin A is a circulating cachexia‐promoting factor in CKD that can cause skeletal muscle atrophy through the soluble activin receptor IIB [10]. In another studies, administration of circulating TNF and IL‐6 caused insulin resistance, whereas administration of neutralizing agents of these cytokines improved skeletal muscle atrophy [11, 12]. Currently, our understanding of the aetiology and mechanisms of CKD‐related sarcopenia remains limited and requires further research.

Recently, various multiomics research approaches have been widely used to study kidney diseases [13]. Omics methods can provide additional information on kidney disease prediction and prognosis. A proteomic study found that serum amyloid A1 protein may be a candidate biomarker for distinguishing calcineurin inhibitor remission from nonremission in primary membranous nephropathy [14]. Some studies, including urine protein profiles of numerous patients, have revealed that CKD273 may be more practical than standard indicators for detecting and predicting CKD [15]. Omics methods can also be used to explore the pathogenesis of kidney diseases. Single cell RNA sequencing (scRNA‐seq) after ischemia–reperfusion injury showed that ferroptosis is crucial for renal tubular cells. The pharmacological targeting of ferroptosis may provide a potential strategy for treating ischemic kidney injury [16].

Based on the development of multiomics technology, this study explores the relationship between the kidney and muscles in primary diseases combined with multiomics. However, it is difficult to obtain kidney, blood and skeletal muscle samples simultaneously in patients with CKD. Diet‐induced models of uraemia and sarcopenia are noninvasive, economical and convenient. Therefore, we induce this model by feeding mice with a diet containing 0.2% adenine for 6 weeks [17]. Next, we establish a regulatory network of kidney‐muscle crosstalk by kidney transcriptome and protein profiles of kidney, serum and muscle to explore the potential mechanism of CKD‐related sarcopenia. Finally, we validated the sequencing results using myotubes in vitro and neutralization antibody experiments in animal models.

## 2 Materials and Methods

### 2.1 Animals

Eight‐week‐old male C57BL/6JNifdc mice were purchased from Vital River Laboratories (Beijing, China). After 2 weeks of adaptive feeding, the mice were randomly divided into two groups, and there was no statistically significant difference in body weight between the two groups (Normal Control [NC] group vs. CKD group, n = 6 per group). An established adenine diet model was used to induce CKD in mice [5]. NC mice or CKD mice were fed with an AIN‐93G diet or a 0.2% adenine AIN‐93G diet (Dyets, Bethlehem Pennsylvania, USA) for 6 weeks. Table S1 presents detailed information about the diet composition. The body weight and grip strength of the mice were measured once per week. At week 6, the kidney, serum, skeletal muscle (gastrocnemius, tibialis anterior and soleus), liver, heart, pancreas, spleen and lung were collected for the corresponding detection.

For the Spp1 neutralization test, 10‐week‐old male C57BL/6JNifd mice were fed with a 0.2% adenine diet for 4 weeks. Subsequently, all mice were randomized to receive intraperitoneal injections of either phosphate‐buffered saline with normal IgG or Spp1 neutralizing antibody (100 μg/kg/day) once a day for two consecutive weeks. At week 6, the skeletal muscle (gastrocnemius, tibialis anterior and soleus) and serum were collected for corresponding detection.

Animal care and experimental procedures were approved by the Ethics Committee on Animal Research of the Jinling Hospital (Approval No. 2022DZGKJDWLS‐00168).

### 2.2 Patients

Patients with renal cancer who underwent radical nephrectomy were enrolled at Jinling Hospital. The inclusion criteria were as follows: (1) age ≥ 18 years, regardless of sex; (2) diagnosis of CKD (eGFR< 60 mL/min/1.73 m^2^, ≥ 3 months) or not (eGFR > 90 mL/min/1.73 m^2^, as controls); and (3) voluntarily agree to participate in this study. The exclusion criteria were as follows: (1) acute kidney injury (AKI) or failure, (2) active infection, (3) pregnancy, (4) combined with hepatitis B, syphilis, HIV infection. Kidney samples far from the tumour site were obtained from these patients. This study conformed to the ethical guidelines of the 1975 Declaration of Helsinki and was approved by the Ethics Committee of Jinling Hospital, written informed consent was obtained from each patient.

### 2.3 Grip Strength Measurement

A mouse grip testing instrument (Nanjing Calvin Biotechnology Co., LTD, Nanjing, China) was used to measure the grip strength of the mouse limbs every week, with an interval of 30 min. The maximum of the three grip strengths was considered the final result.

### 2.4 Assessment of Kidney Function

Blood urea nitrogen (BUN) and serum creatinine (Scr) levels were measured using Nanjing Jiancheng Biological Engineering urea and creatinine kits (Nanjing, China), in accordance with the manufacturer's instructions.

### 2.5 Morphological Analysis of Kidney and Muscle Sections

The mouse kidneys and gastrocnemius muscles were fixed in 4% paraformaldehyde and embedded in paraffin. The kidneys were stained with haematoxylin and eosin (HE), Masson's trichrome and periodic acid–Schiff (PAS). Gastrocnemius muscle was further processed for HE and Sirius Red staining. Slices were analysed using an upright fluorescence microscope (Zeiss, Germany).

### 2.6 RNA Sequencing

RNA‐Seq analysis of kidneys from CKD mice was performed using LC‐Bio (Hangzhou, China) according to the manual, and RNA‐Seq analysis of the gastrocnemius muscle was performed after pharmacological inhibition of Spp1 using Novogene (Beijing, China) according to the manual. Details of RNA sequencing are provided in the Supporting Information.

### 2.7 Mass Spectrum by Tandem Mass Tag (TMT)

In the kidney and gastrocnemius tissues (CKD vs. NC, n = 4 per group), protein was extracted by homogenate and sodium dodecyl sulphate lysis [18]. For the serum (CKD vs. NC = 4 vs. 4), we utilized the immunoaffinity depletion of serum high‐abundance proteins. We used SDS‐PAGE separation, filter‐aided sample preparation [19], TMT labelling, peptide fractionation with reversed‐phase chromatography and mass spectrometry analysis. Mass spectrometry analyses included nLC, LC‐MS/MS and data analysis. Differentially expressed proteins (DEPs) between the two groups were determined using the *t*‐test (for serum and kidney tissue, the threshold values were set as *p* < 0.05, and 1.5‐fold change; for gastrocnemius tissue, the threshold values were set as *p* < 0.05, and 1.2‐fold change).

### 2.8 Kyoto Encyclopedia of Genes and Genomes (KEGG) and Gene Set Enrichment Analysis (GSEA) Analysis

KEGG pathway analysis was performed using DEG/DEPs and drawn at the Lianchuan Biological Cloud Platform (https://www.omicstudio.cn/home). An adjusted *p* value (*q* value) < 0.05 was used for the KEGG pathway of gastrocnemius TMT, and a *p* value < 0.05 was used for the KEGG pathway of gastrocnemius RNA‐seq. The enrichment factor was the ratio between the sequenced gene and all annotated genes enriched in the pathway. GSEA has been extensively applied to identify the underlying pathways [20]. The canonical pathways (KEGG subset) collection was obtained from the molecular signatures database (MsigDB). *p* values < 0.05 and |normalized enrichment score (NES)| > 1 were considered statistically significant.

### 2.9 Establishment of Correlation Network Diagram

Correlation analysis was performed between the abundance of focal proteins in serum TMT and the abundance of DEPs in the KEGG pathway from muscle TMT. We performed person correlation analysis using SPSS26.0 (IBM, Chicago, IL, USA) to obtain correlation coefficients and *p* values. The above results of *p* < 0.05 were uploaded to the Lianchuan Biological Cloud Platform (https://www.omicstudio.cn/home) for drawing correlation network diagrams.

### 2.10 Cell Culture

Mouse C2C12 myoblasts were obtained from the Chinese Academy of Science Cell Bank. Myoblasts were cultured in DMEM/HG (Gibco, New York, USA) supplemented with 10% foetal bovine serum (FBS) (Gibco) and 1% penicillin–streptomycin (KeyGEN BioTECH, Nanjing, China) at 37°C and 5% CO_2_. When the cells were confluent to 80%–90%, the culture medium was replaced wtih a differentiation medium consisting of DMEM/HG and 2% horse serum (HS, Gibco). After 4 days, different concentrations (0, 10, 100, and 1000 ng/mL) of Spp1(abcam, Cambridge, UK) and S100a9 (SinoBiological, Nanjing, China) recombinant proteins were treated for 48 h. Thereafter, the myotubes were harvested for corresponding detection. Three independent experiments were performed for each condition.

### 2.11 Western Blotting

Proteins were extracted from C2C12 cells, kidney and gastrocnemius muscles of mice with RIPA lysis buffer (Beyotime, Shanghai, China) on ice. We added protein samples to 10% SDS polyacrylamide gels, subjected to electrophoresis (at constant pressure, 70 V for 30 min, 140 V for 50 min), transferred to PVDF membranes (Millipore, Billerica, MA, USA) under a constant current of 300 mA for 90 min, and blocked with 5% milk in 1× TBST for 90 min. Next, the membranes were incubated using the following primary antibodies at 4°C overnight: anti‐Fbx32 (Atrogin‐1) antibody, Murf‐1 antibody and Spp1 antibody. GAPDH was used as a reference standard. The next day, the PVDF membranes were incubated with a horseradish peroxidase (HRP)‐conjugated secondary antibody for 1.5 h. Finally, the immunoblots were visualized with an enhanced chemiluminescence (ECL) HRP substrate Kit (Millipore, Billerica, MA, USA) and captured in a Tanon 4600 series automatic chemiluminescence image analysis system (Tanon, Shanghai, China). The antibodies used are listed in Table S2.

### 2.12 Enzyme‐Linked Immunosorbent Assay (ELISA)

The concentrations of Spp1 in mouse serum were measured using commercially available ELISA kits (abcam, Cambridge, UK), according to the manufacturer's protocol.

### 2.13 Immunohistochemistry

Immunohistochemical staining was performed on mouse kidney tissues and human kidney biopsy tissues. For the immunohistochemical assays, paraffin‐embedded sections of mouse and human kidneys, cut into 4‐μm‐thick slices, were deparaffinized and hydrated using Antigen Retrieval Buffer solution (abcam, Cambridge, UK). Endogenous streptavidin activity was blocked using 3% hydrogen peroxide. The sections were stained with Spp1 antibody and then incubated with the appropriate HRP‐conjugated secondary antibody (goat anti‐mouse IgG). Finally, all sections were counterstained with Mayer's haematoxylin (Servicebio, Wuhan, China) and evaluated under the upright fluorescence microscope (Zeiss, Germany). The antibodies used are listed in Table S2.

### 2.14 Quantitative Real‐Time (q‐RT) PCR Analysis

Total RNA from the liver, skeletal muscle, heart, kidney, spleen, lung and pancreatic tissues was extracted using TRIzol Reagent (Invitrogen, California, USA) and RNA Extraction Kit (Tiangen, Beijing, China), according to the manufacturer's instructions. The RNA concentration was measured using a NanoDrop instrument (Agilent Technologies, USA), followed by the synthesis of single‐stranded cDNA using HiScript III RT SuperMix for qPCR (Vazyme, Nanjing, China), according to the manufacturer's instructions. Finally, the SYBR Green Master Mix (Vazyme, Nanjing, China) and ABI SteponePlus PCR system (Thermo Fisher Scientific, USA) were used for relative quantification. Fold changes in the expression of target genes were calculated using the 2^−ΔΔCT^ method, with GAPDH used as an endogenous reference. The primers employed were synthesized by Invitrogen (California, USA) and are listed in Table S3.

### 2.15 Immunofluorescence Staining

C2C12 myotubes were fixed in 4% paraformaldehyde for 15 min. Next, antigen blockage with 3% BSA sealing were performed. Subsequently, the myotubes were stained with primary antibodies against MYH antibody at 4°C overnight. The myotubes were incubated with fluorescent‐conjugated secondary antibody in the dark for 1 h and then counterstained with DAPI (1:500, Invitrogen, California, USA). Finally, the myotubes were photographed with an inverted fluorescence microscope (CarlZeiss, Werk Gottingen, Germany). The fluorescence intensity was analysed by using Image J 1.52a software (National Institutes of Health, USA). The antibodies used are listed in Table S2.

### 2.16 Statistical Analysis

Data were analysed with Graphpad Prism 5 (San Diego, CA, USA) and expressed as mean ± standard deviation (SD). The means for two group data were compared by the unpaired *t*‐test. Differences across condition were analysed using one‐way repeated measures analysis of variance (ANOVA), with Bonferroni post‐hoc analysis used to detect where interaction effect lay within the data set. Statistical significance occurred if a computed two‐tailed *p* < 0.05.

## 3 Results

### 3.1 Establishment of a Mouse Model of CKD‐related Sarcopenia

**FIGURE 1:**
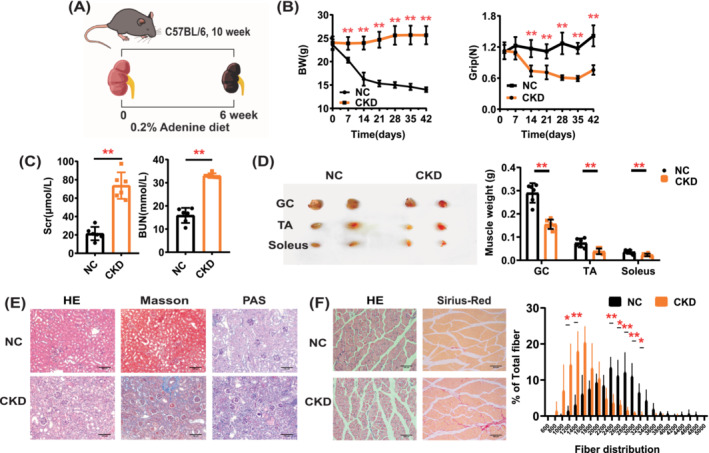
Establishment of a mouse model of CKD‐related sarcopenia. (A) Schematic representation of CKD model: mice are fed for 6 weeks with adenine‐enriched diet. (B) Body weight and grip curve in NC and CKD mice (*n* = 6 mice). (C) BUN and Scr measurement (*n* = 6 mice). (D) Representative images of gastrocnemius (GC), tibialis anterior (TA) and soleus muscles and differences in weight in NC and CKD mice (*n* = 6 mice). (E) Representative images of renal pathology (HE staining, Masson staining and PAS staining) in NC and CKD mice. (I) Representative pathological images of GC muscle and frequency histogram showing the distribution of cross‐sectional areas (μm^2^) in GC muscle of NC and CKD mice (*n* = 3 mice). Data are expressed as mean ± SD. *p < 0.05, **p < 0.01. Scale bars: 100 μm.

After feeding 10‐week‐old C57BL/6JNifdc mice with a 0.2% adenine diet for 6 weeks (Figure 1A), there was a progressive decrease in body weight and grip strength and a marked increase in BUN and Scr (Figure 1B,C, Figure S1). Renal pathology analysis showed significant tubular atrophy and interstitial fibrosis in CKD mice (Figure 1E). As shown in Figure 1D, the weights of the gastrocnemius, tibialis anterior and soleus muscles were significantly reduced in CKD mice compared with those in the NC group. The pathology and cross‐sectional area (CSA) analysis of the muscle showed that the CSA was dramatically reduced in CKD mice (Figure 1F). Our results showed that 0.2% adenine significantly reduced kidney function and increased skeletal muscle loss in mice.

### 3.2 Transcriptome and Protein Mass Spectrum Analysis Identified the DEG/DEPs Derived From Kidney Tissue and Serum Between CKD and NC Mice

**FIGURE 2:**
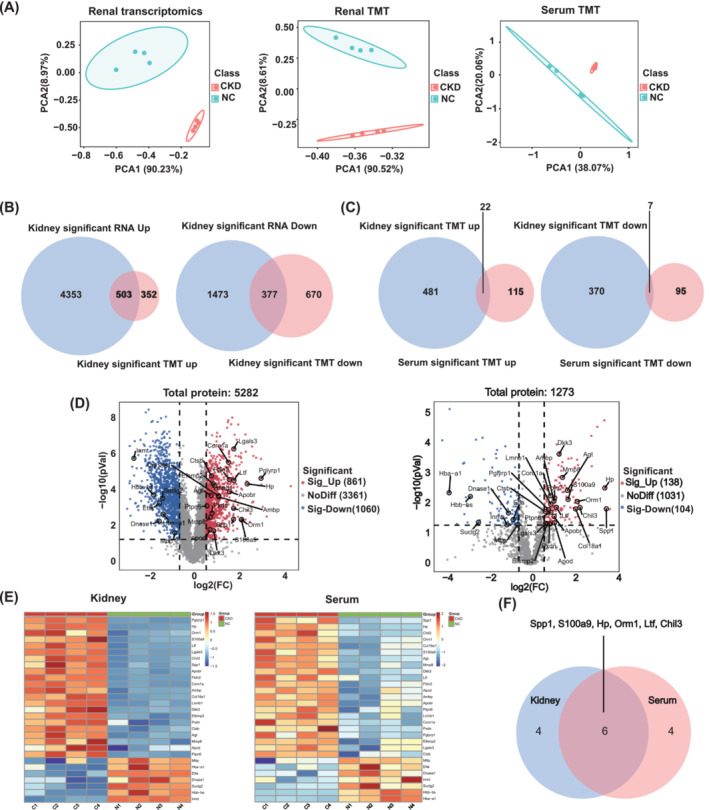
Transcriptome and protein mass spectrum analysis identified the DEG/DEPs derived from kidney tissue and serum between CKD and NC mice. (A) PCA of renal transcriptome, renal TMT and serum TMT. (B) Venn diagram showing the co‐upregulated/co‐downregulated DEPs/DEGs between renal transcriptome and TMT. (C) Venn diagram showing co‐upregulated (*n* = 22)/downregulated (*n* = 7) DEPs between renal transcriptome/TMT and serum TMT. (D) Volcanic plot of renal TMT and serum TMT (29 proteins in Figure 2C were labelled). (E) Heatmaps showing the levels of 29 proteins in the TMT of kidney and serum; each row represents one protein, and each column represents one sample. (F) Venn intersection of the top 10 proteins expressed in CKD kidney and serum from Figure 2E.

The pathogenesis of sarcopenia is complex; this process cannot be studied in isolation [50]. Renal dysfunction is the primary cause of skeletal muscle loss in patients with CKD. Therefore, we first performed the transcriptome and TMT of kidney tissues and TMT of serum in CKD and NC mice. The transcriptome and TMT of kidney and TMT of serum clearly segregated CKD and NC mice based on principal component analysis (PCA) (Figure 2A). Combined with differentially expressed genes (DEG)/DEPs from renal transcriptome and TMT analysis, a total of 503 genes/proteins were co‐upregulated and 377 proteins/genes were co‐downregulated in the CKD groups compared with the control (Figure 2B). Subsequently, the co‐upregulated and co‐downregulated proteins/genes were overlapped with the upregulated and downregulated proteins of serum TMT, respectively. Finally, 22 upregulated and 7 downregulated proteins were identified (Figure 2C). Volcanic figures and heat maps showed 29 DEGs/DEPs in the kidney and serum TMT between the NC and CKD groups (Figure 2D,E). After overlapping the top 10 most expressed proteins in the CKD kidney and serum from Figure 2E, we identified 6 proteins, including Spp1, S100a9, Hp, Orm1, Ltf and Chil3 (Figure 2F). Our study is the first to describe a DEPs atlas derived from the kidney/serum under CKD state, providing potential therapeutic targets for CKD‐induced sarcopenia. These observations encouraged us to investigate the function of the putative protein in CKD‐induced skeletal muscle loss.

### 3.3 TMT Data Revealed Molecular Alterations of Gastrocnemius Muscle in Mice With CKD

**FIGURE 3:**
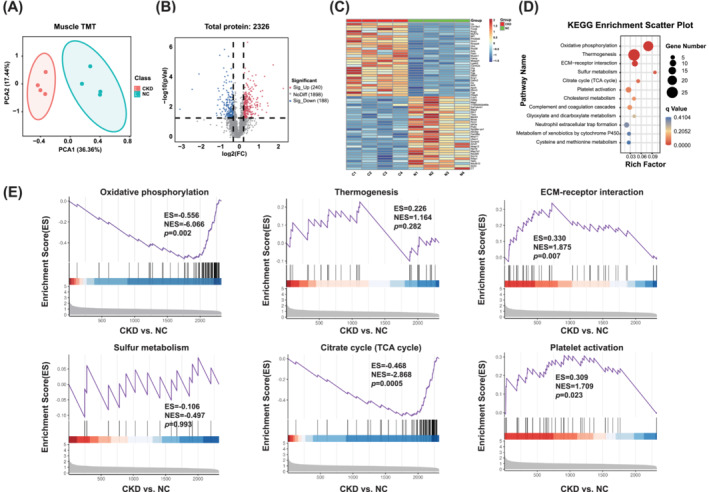
TMT data revealed molecular alterations of gastrocnemius muscle in mice with CKD. (A) PCA of gastrocnemius muscle TMT. (B) Volcanic plot of gastrocnemius muscle TMT. (C) Heatmap represented the top 30 DEPs among the upregulated and downregulated proteins in the gastrocnemius muscle of CKD and NC mice. (D) KEGG enrichment scatter plot represented the significantly altered pathways. (E) GSEA plots of oxidative phosphorylation signalling pathway, thermogenesis pathway, ECM‐receptor interaction pathway, sulphur metabolism, Citrate cycle (TCA cycle) and platelet activation in gastrocnemius of CKD and NC mice, respectively.

TMT proteomics of the gastrocnemius muscle was performed to further investigate molecular alterations in CKD‐induced sarcopenia. The TMT segregated the gastrocnemius muscle of CKD and NC mice based on the PCA (Figure 3A). The volcano plot revealed that 2326 proteins were detected between CKD and NC mice, of which 240 and 188 proteins were significantly upregulated and downregulated, respectively (Figure 3B). The heatmap represents the top 30 DEPs among the upregulated and downregulated proteins in the gastrocnemius muscles of CKD mice (Figure 3C). The rich factor‐based KEGG pathway enrichment analysis displayed that pathways were significantly altered, including oxidative phosphorylation signalling pathway, thermogenesis pathway, ECM‐receptor interaction pathway, sulphur metabolism, citrate cycle (TCA cycle) and platelet activation (Figure 3D). We then performed GSEA for these six pathways to explore the molecular changes in CKD with sarcopenia. Notably, GSEA of TMT data from the gastrocnemius muscle in mice with CKD showed that oxidative phosphorylation and TCA cycle pathways were significantly downregulated, whereas ECM‐receptor interaction and platelet activation pathways were significantly upregulated (Figure 3E). These results indicate that the dysregulation of proteins involved in the above pathways are closely related to CKD‐induced sarcopenia.

### 3.4 Correlation Network Diagram Between Renal‐Secreted Proteins and Proteins Invovled in the Most Significantly Altered KEGG Pathways in Skeletal Muscle

**FIGURE 4:**
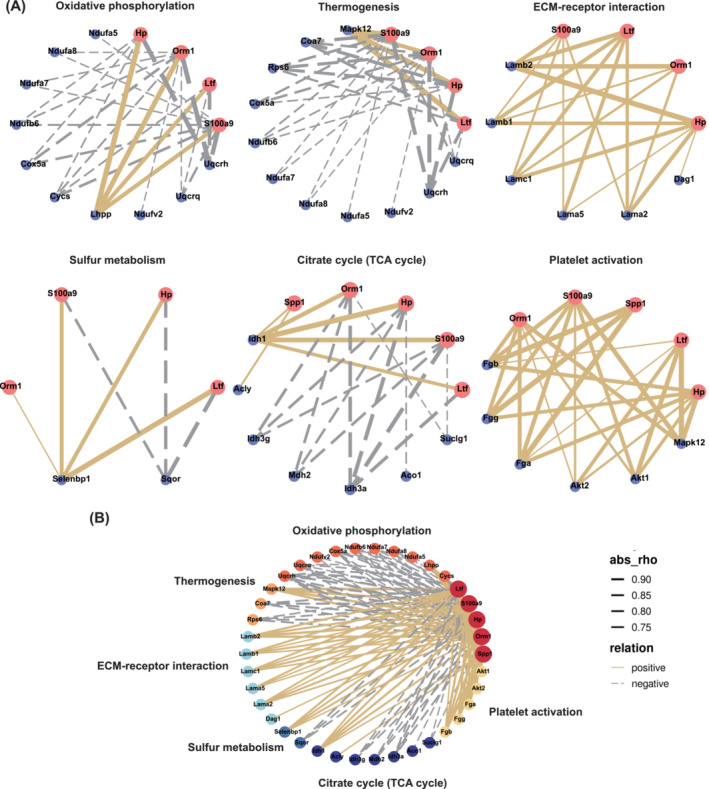
Correlation network diagram between renal‐secreted proteins and proteins on the most significantly altered KEGG pathways in skeletal muscle. (A) The correlation networks of five renal‐secreted proteins and proteins with significant differences in the oxidative phosphorylation signalling pathway, thermogenesis pathway, ECM‐receptor interaction pathway, sulphur metabolism, citrate cycle (TCA cycle) and platelet activation pathway in skeletal muscle, respectively. (B) A summary of all pathways in Figure 4A. abs: absolute value; rho: correlation coefficient. The thickness of the line indicates the absolute value of the correlation coefficient. Relationships were classified as positive and negative.

We hypothesized that the kidneys secrete specific proteins into the blood in the CKD state and that these proteins may cause skeletal muscle loss via blood circulation. We performed a heatmap of the correlation between the expression level of the screened 29 proteins with Scr, BUN, gastrocnemius muscle weight and grip strength (Figure S2). Combined with the correlation heatmap, we further narrowed the six proteins in Figure 2F to five: Spp1, S100a9, Hp, Orm1 and Ltf (Chil3 was excluded because it was only correlated with gastrocnemius muscle). A correlation network analysis between these five proteins in serum TMT and the proteins involved in the top six KEGG pathways of muscle TMT was performed to explore the potential effect of these proteins on muscle function. The expression levels of S100a9, Hp, Orm1 and Ltf in serum TMT were negatively correlated with the changes of most DEPs in oxidative phosphorylation and thermogenesis pathways in muscle TMT but were positively correlated with DEPs in the ECM‐receptor interaction pathway in muscle TMT (Figure 4A). Spp1, S100a9, Hp, Orm1 and Ltf levels in serum TMT were positively correlated with most DEPs in TCA cycle and the platelet activation pathway in the muscle TMT (Figure 4A). S100a9, Hp, Orm1 and Ltf in serum TMT were negatively correlated with Selenbp1 but positively correlated with Sqor in sulphur metabolism in muscle TMT (Figure 4A). These results elucidate the pathways in which the target proteins may be involved.

### 3.5 Effect of Spp1 and S100a9 Recombinant Protein on the Myotube Atrophy In Vitro

**FIGURE 5:**
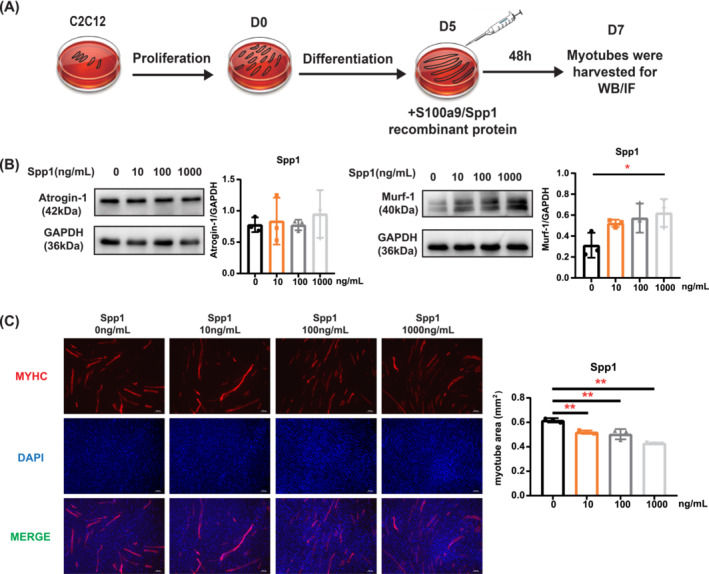
Effect of Spp1 recombinant protein on the myotube atrophy in vitro. (A) Flow chart of C2C12 cell culture. (B) Western blot analysis of atrogin‐1 and murf‐1 treated with different concentrations (0, 10, 100, and 1000 ng/mL) of recombinant protein Spp1. (C) Immunofluorescence staining of myosin heavy chain (MHC) treated with different concentrations (0, 10, 100, and 1000 ng/mL) of recombinant protein Spp1. Data are expressed as mean ± SD of three independent experiments. Statistical significance was evaluated using a one‐way ANOVA test. Scale bars: 100 μm. *p < 0.05, **p < 0.01.

After screening and literature review, two proteins, Spp1 and S100a9, became our first focus targets. Spp1 and S100a9 recombinant proteins were used to study their effects on C2C12 myotubes. Figure 5A shows a flowchart of the cell experiment. After adding S100a9 recombinant protein (100 ng/mL), the protein level of atrogin‐1 was significantly increased (Figure S3A). However, no difference was observed in atrogin‐1 protein level after adding different concentrations of Spp1 recombinant protein (Figure 5B). The murf‐1 protein levels significantly increased after adding 1000 ng/mL Spp1 and S100a9 recombinant proteins, respectively (Figure 5B, Figure S3A). The immunofluorescence staining indicated that high concentrations of Spp1 or S100a9 recombinant proteins reduced myotube area (Figure 5C, Figure S3B). Thus, Spp1 and S100a9 are associated with the molecular phenotypes of sarcopenia.

### 3.6 Validation Analysis of Spp1 in Kidney, Serum and Skeletal Muscle in the CKD Mouse Model

**FIGURE 6:**
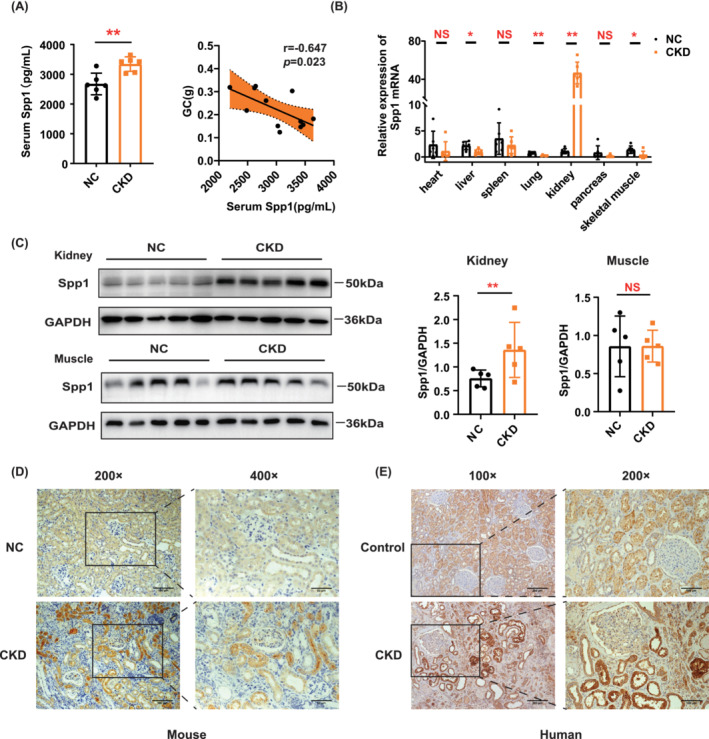
Validation analysis of Spp1 in kidney, serum and skeletal muscle in the CKD mouse model. (A) Left panel: Serum level of Spp1 in NC and CKD mice determined by ELISA (*n* = 6 mice). Right panel: Pearson correlation analysis of Spp1 serum level and gastrocnemius (GC) muscle weight (*n* = 6 mice). (B) Quantitative reverse transcription PCR (RT‐PCR) analysis of Spp1 in different organs (*n* = 6 mice). (C) Western blot analysis of Spp1 in kidney and skeletal muscle of NC and CKD mice (*n* = 5 mice). (D) Representative images of Spp1 immunohistochemistry in NC and CKD mice. Scale bars: 50 μm and 100 μm. (E) Representative images of Spp1 immunohistochemistry in healthy controls and CKD patients. Scale bars: 100 μm and 200 μm. Data are expressed as mean ± SD. *p < 0.05, **p < 0.01.

Among these two targets, we selected Spp1 for further verification. We observed an increase in serum Spp1 concentrations in CKD mice (Figure 6A), which were negatively correlated with skeletal muscle mass (*r* = −0.647, *p* = 0.023, Figure 6A). qRT‐PCR indicated that Spp1 mRNA levels were significantly increased in the kidney of CKD mice (Figure 6B), which was further confirmed by WB and immunohistochemistry (Figure 6C,D). Simultaneously, Spp1 expression levels in the kidneys of CKD patients were significantly increased by immunohistochemistry (Figure 6E). We also examined Spp1 expression in skeletal muscle using WB and found no difference between NC and CKD mice (Figure 6C). Thus, kidney‐produced Spp1 may affect skeletal muscle mass via blood circulation.

### 3.7 Pharmacological Inhibition of Spp1 Prevented Muscle Wasting in Experimental CKD

**FIGURE 7:**
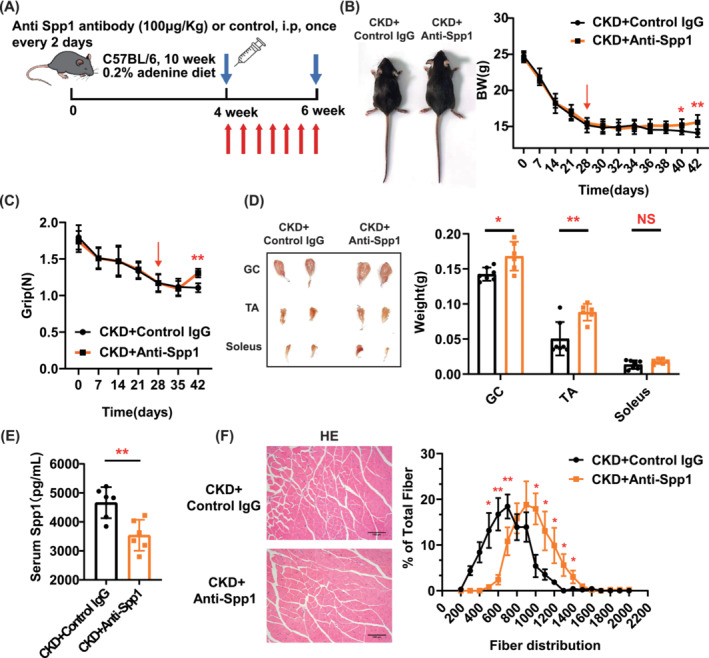
Pharmacological inhibition of Spp1 prevents muscle wasting in experimental CKD. (A) Schematic representation of CKD mice receiving an intraperitoneal injection of Control IgG (*n* = 7 mice) and anti‐Spp1 antibody (*n* = 6 mice). (B) Representative image and body weight curve in receiving control IgG (*n* = 7 mice) and anti‐Spp1 antibody mice (*n* = 6 mice). (C) Grip was measured in receiving control IgG (*n* = 7 mice) and anti‐Spp1 antibody mice (*n* = 6 mice). (D) Representative images of gastrocnemius (GC), tibialis anterior (TA) and soleus muscles and differences in weight in receiving control IgG (*n* = 7 mice) and anti‐Spp1 antibody mice (*n* = 6 mice). (E) Blood level of Spp1 in receiving control IgG and anti‐Spp1 antibody mice determined by ELISA (*n* = 6 mice). (F) Representative pathological images of GC muscle and frequency histogram showing the distribution of cross‐sectional areas (μm^2^) of GC muscle in receiving control IgG and anti‐Spp1 antibody mice (*n* = 4 mice). Data are expressed as mean ± SD. *p < 0.05, **p < 0.01. Scale bars: 100 μm.

Considering that increased blood levels of Spp1 are a hallmark of CKD mice, we pharmacologically inhibited Spp1 using Spp1‐neutralizing antibodies. After a 4‐week 0.2% adenine diet, CKD mice received Spp1‐neutralizing antibodies once every other day by intraperitoneal injections (100 μg/kg) for two consecutive weeks (Figure 7A). Treatment with Spp1 neutralizing antibody prevented body weight loss in CKD mice (Figure 7B). After 2 weeks of Spp1‐neutralizing antibody treatment, grip strength was significantly higher in the anti‐Spp1 group than in the control IgG group (Figure 7C). Simultaneously, we observed that the weights of gastrocnemius muscle and tibialis anterior muscle in anti‐Spp1 group mice were greater than those of control IgG group (Figure 7D). The weight of the soleus muscle was not prevented by Spp1‐neutralizing antibody (Figure 7D). Neutralization of circulating Spp1 reduced the blood Spp1 concentration and mildly improved serum urea nitrogen and creatinine levels in CKD mice (Figure 7E, Figure S4A,B). Quantification of CSA of gastrocnemius muscles showed a rightward shift at the terminal time point in Spp1 neutralizing antibody compared with control IgG mice (Figure 7F). Therefore, we found that pharmacological inhibition of Spp1 prevents muscle wasting in experimental CKD.

### 3.8 Transcriptome Analysis Identified the DEGs Derived From Gastrocnemius Muscle After Pharmacological Inhibition of Spp1 and Validation of Skeletal Muscle Atrophy Markers

**FIGURE 8:**
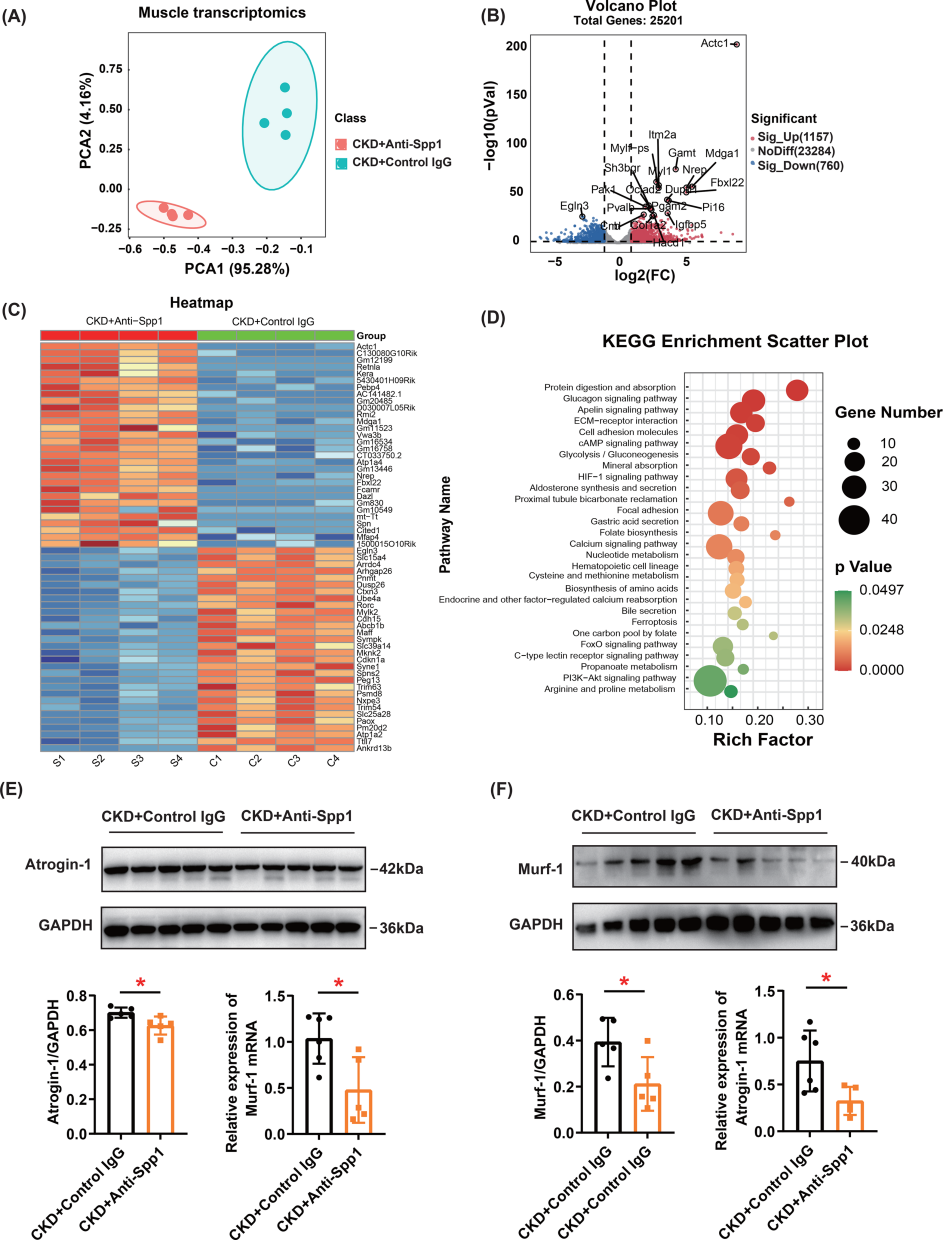
Transcriptome analysis identified the DEGs derived from gastrocnemius muscle after pharmacological inhibition of Spp1 and validation of skeletal muscle atrophy markers. (A) PCA of skeletal muscle transcriptome. (B) Volcanic plot of skeletal muscle transcriptome. (C) Heatmap represented the top 30 DEGs among the upregulated and downregulated genes in the gastrocnemius muscle. (D) KEGG enrichment scatter plot represented the significantly altered pathways. (E,F) Western blot and quantitative reverse transcription PCR (RT‐PCR) analysis of Atrogin‐1 and Murf‐1 in the gastrocnemius muscle of control IgG and Anti‐Spp1 antibody mice. Data are expressed as mean ± SD. *p < 0.05, **p < 0.01.

To identify the DEGs influenced by Spp1 inhibition in CKD mouse muscles, we performed a transcriptomic analysis comparing anti‐Spp1 and control IgG‐treated GC muscles in CKD mice. The RNA sequencing segregated the gastrocnemius muscle of anti‐Spp1 and control IgG group according to PCA (Figure 8A). The volcano plot revealed that 25 201 genes were detected between these two groups, of which 1157 and 760 genes were significantly upregulated and downregulated, respectively (Figure 8B). The heatmap represented the top 30 DEGs among the upregulated and downregulated genes in the gastrocnemius muscle of CKD mice after receiving Spp1 neutralizing antibody treatment (Figure 8C). The rich factor‐based KEGG pathway enrichment analysis showed that the 30 significantly altered pathways, among which protein digestion and absorption, glucagon signalling pathway, apelin signalling pathway, ECM‐receptor interaction and cell adhesion molecules were the most dramatically altered (Figure 8D). Simultaneously, we verified the expression of skeletal muscle atrophy markers in the gastrocnemius muscles after treatment with Spp1‐neutralizing antibody. We found that pharmacological inhibition of Spp1 improved markers of muscle atrophy (atrogin‐1 and murf‐1), both at the mRNA and protein levels (Figure 8E,F). These data provide clues to the mechanism by which blockade of circulating Spp1 increases skeletal muscle mass.

## 4 Discussion

To the best of our knowledge, this is the first study to perform kidney transcriptome and protein profiling of kidney, serum and muscle protein profiling in CKD and NC mice. First, we created a mouse model containing 0.2% adenine. Next, a regulatory network of ‘kidney‐muscle’ crosstalk was established using multiomics analysis to explore the potential mechanism of CKD‐related sarcopenia. Finally, we validated the sequencing results using cell and neutralization antibody experiments in animal models. The study expounded the new intervention targets from the perspective of the kidney‐muscle axis and provided a theoretical basis for improving the prognosis and quality of life of patients with CKD.

The combined analysis of the same omics from different tissues or omics from the same tissue in a disease model is a commonly used method for multiomics combined analysis [21, 22]. In this study, various DEPs/DEGs were identified in the kidney transcriptome and protein profiles of CKD mice. However, only 22 proteins were simultaneously upregulated with serum TMT in CKD mice. Among these 22 proteins, well‐known kidney injury markers such as Dkk3, Lgals3 and Agt [21, 23, 24] were observed in the renal TMT of CKD mice, indicating that our animal model was as successful as previous studies in reflecting chronic kidney injury. Certain proteins (Coro1a1 and S100a9) have been indicated to control of myeloid cell apoptosis and proliferation [25]. Among the remaining proteins, some are associated with chronic inflammation in kidney disease (Chil3 and Mmp8) [26], others are components of the glomerular basement membrane (Col18a1) [27], and others have an uncertain function in kidney disease.

To further identify the circulating factors associated with sarcopenia in CKD, we selected five kidney/serum‐derived (Spp1, S100a9, Orm1, Hp and Ltf) proteins from the 22 proteins based on their high fold‐change and correlation with indicators of CKD and sarcopenia. Martin et al. performed a plasma proteomic study using the iTRAQ method in hypertensive patients with different albuminuria levels and found that Hp and Orm1 were upregulated in hyperalbuminuria [28]. Orm1 and Hp are principally produced by the liver, and the elevated plasma levels of HP and Orm1 in patients with CKD may be a hepatic compensatory mechanism to counteract the loss of proteinuria [28, 29, 30]. Ltf is an antioxidant, antibacterial and anti‐inflammatory factor that provides an innate defence against a range of damaging stimuli [31]. The elevation of Ltf in the kidney and serum TMT levels in our study may represent renal repair mechanisms in response to persistent injury. Among the remaining proteins, S100a9 and Spp1 have clear functions in kidney disease. However, it is unclear whether these two proteins act as circulating factors that cause skeletal muscle wasting under CKD conditions.

Our kidney transcriptome and proteome sequencing results from CKD mice showed significantly increased expression of Spp1, which is consistent with previous reports. A single‐cell sequencing study specifically showed that Spp1 is secreted by injured proximal tubules, podocytes and fibroblasts in a diabetic nephropathy model [32]. Another proteomic study showed that Spp1 expression significantly increased in the kidneys of mice with folic acid induced AKI [33]. Osteopontin (OPN), encoded by Spp1, is expressed in numerous cells, including hepatocytes, osteoclasts, osteoblasts, renal epithelial cells, fibroblasts and renal tubular cells [34], and is essential for regulating inflammation and immunity [34]. Numerous studies have shown that OPN expression was increased in the kidneys, blood and urine of patients with CKD (diabetic nephropathy, glomerulonephritis and immunoglobulin A nephropathy), suggesting that OPN might be a biomarker for CKD [35, 36]. In our 0.2% adenine‐induced CKD mouse model, serum Spp1 concentration was elevated (Figure 6A), as well as the protein and mRNA expression of Spp1 in the kidneys (Figure 6B, C), which is consistent with previous reports.

The regulatory role and mechanism of action of OPN in skeletal muscle are not uniform. Current evidence suggests that OPN expression is required for normal muscle regeneration after a single severe injury [37, 38]. However, increased OPN may be a vital factor in the decline of muscle repair capacity in older adults [39, 40]. Overexpression of OPN may lead to chronic damage, fibrosis and dysfunction in dystrophic muscles [41]. Our results showed that the mRNA levels of Spp1 were decreased in skeletal muscle of CKD mice, but WB analysis indicates that there is no change in the protein levels (Figure 6B,C). Recently, Khamissi et al. reported that circulating OPN released from damaged kidneys triggers respiratory failure and found that OPN may act on CD44‐expressing macrophages in the lungs [42]. Based on this literature review, we hypothesized that an increase in circulating Spp1 levels under CKD conditions induces skeletal muscle atrophy. Therefore, we intraperitoneally injected Spp1 neutralizing antibody into CKD mice. The results showed a decrease in serum Spp1 concentration and an increase in skeletal muscle mass. To identify the DEGs influenced by Spp1 inhibition in CKD mouse muscles, we performed a transcriptomic analysis comparing anti‐Spp1 and control IgG‐treated GC muscles in CKD m‐ice. The rich factor‐based KEGG pathway enrichment analysis showed that protein digestion and absorption, glucagon signalling pathway, apelin signalling pathway, ECM‐receptor interaction, cell adhesion molecules were the most dramatically altered signalling pathways (Figure 8D). However, the exact mechanism underlying Spp1‐induced skeletal muscle atrophy requires further investigation.

In our cellular experiments, in addition to Spp1, we found that S100a9 was associated with the molecular phenotype of muscle atrophy. S100a8 and S100a9 belong to the S100a family of calcium‐and zinc‐binding proteins, which play prominent roles in regulating inflammatory processes and immune responses [43, 44]. A scRNA‐seq study of the mononuclear phagocytic system in an AKI mouse model (ischemia reperfusion injury) identified a population of S100a9^hi^‐Ly6c^hi^ infiltrated macrophages as early responders to AKI, mediating initiation and expansion of renal inflammation [45]. Another study found that S100a8 and S100a9 were upregulated in the kidney proteome of mice with diabetic nephropathy (DN) [46]. In addition, S100a8/a9 levels were found to be elevated in human advanced DN kidneys by GEO analysis (GSE142025 and GSE47185). The high‐level expression of S100a8/a9 activates the TLR4/NF‐κB signalling pathway, which promotes the epithelial‐to‐mesenchymal transition process and ultimately leads to renal interstitial fibrosis (RIF) progression [46]. However, the role of S100a9 in sarcopenia has not been well studied. Only one study has reported that S100a9 as a potential pathogenic factor for pancreatic cancer–induced cachexia in vitro [47]. The role of S100a9 on skeletal muscle is required for further validation by more comprehensive functional experiments.

KEGG and GSEA analyses of skeletal muscle TMT revealed that oxidative phosphorylation (OXPHOs), ECM–receptor interaction and TCA cycle were associated with skeletal muscle loss. Mitochondria are critical organelles in the metabolism state of skeletal muscles. Consistent with our results, some studies have found that mitochondrial OXPHOs was significantly reduced in the skeletal muscles of CKD mice, and OXPHOS dysfunction was driven by decreased matrix dehydrogenase activity [5, 6]. Indoxyl sulphate (a uremic toxin) can downregulate the TCA cycle, leading to reduce ATP production associated with sarcopenia [48]. Extracellular matrix (ECM) is an essential component of skeletal muscles and plays a major role in force transmission, maintenance and repair of muscle fibres [49]. Excessive accumulation of ECM components, especially collagen in skeletal muscles can cause skeletal muscle fibrosis [49]. Skeletal muscle fibrosis replaces and impairs original muscle fibres, leading to a loss of muscle mass. Skeletal muscle with increased ECM in patients with CKD was characterized by fibrosis, capillary rarefication and loss of muscle stem cells [50]. The transcriptomic analysis comparing anti‐Spp1 and control IgG‐treated GC muscles in CKD mice revealed that the ECM‐receptor interaction was one of the most dramatically altered signalling pathways (Figure 8D). However, the relationship between Spp1, the ECM and sarcopenia requires further investigation.

For the first time, we found that pharmacological inhibition of Spp1 alleviated skeletal muscle wasting in a CKD mouse model. The limitations of this study are as follows. First, the protective effect of the pharmacological inhibition of Spp1 on skeletal muscle atrophy must be validated in multiple CKD models. Second, the relationship between serum Spp1 concentration and skeletal muscle atrophy in patients with CKD requires further refinement. Finally, the exact mechanism underlying Spp1‐induced skeletal muscle atrophy requires further exploration.

In conclusion, our study combined multiomics sequencing to explore the pathogenesis of CKD‐induced sarcopenia from the perspective of the ‘kidney‐muscle’ axis. Through multiomics analysis, cellular evaluation and animal experiments, Spp1 may be a promising target for treating CKD with sarcopenia.

## Conflicts of Interest

The authors declare no conflicts of interest.

## Supporting information


**Table S1.** Diet Composition Details.
**Figure S1.** A representative image of Normal Control (NC) and chronic kidney disease (CKD) group. Left: NC group; Right: CKD group.
**Figure S2.** Heatmap of the correlation analysis between the screened 29 proteins and biochemical measures and phenotypes. BUN: Blood urea nitrogen; Scr: serum creatinine; GC: gastrocnemius. *p < 0.05, **p < 0.01, ***p < 0.001.
**Figure S3.** The effect of S100a9 recombinant protein on the myotube atrophy in vitro. **(A)** Western blot analysis of atrogin‐1 and murf‐1 treated with different concentrations (0, 10, 100, and 1000 ng/mL) of recombinant protein S100a9. **(B)** Immunofluorescence staining of myosin heavy chain (MHC) treated with different concentrations (0, 10, 100, and 1000 ng/mL) of recombinant protein S100a9. Data are expressed the mean ± SD (*n* = 3). Statistical significance was evaluated using a one‐way ANOVA test. Scale bars: 100 μm. *p < 0.05, **p < 0.01.
**Figure S4.** Effect of pharmacological inhibition of Spp1 on serum creatinine and blood urea nitrogen in experimental CKD. Scr: serum creatinine; BUN: blood urea nitrogen.
**Table S2.** Antibody list for all experiments.
**Table S3.** Primer sequence for qPCR.

## Data Availability

The mass spectrometry proteomics data have been deposited to the ProteomeXchange Consortium (https://proteomecentral.proteomexchange.org) via the iProX partner repository with the dataset identifier PXD055324 (CKD mice serum), PXD055325(CKD mice kidney) and PXD055326(CKD gastrocnemius muscle). The raw sequence data have been submitted to the NCBI Gene Expression Omnibus (GEO) datasets with NCBI Short Read Archive (SRA) with accession number with PRJNA1147118 (CKD mice kidney) and PRJNA1151987(gastrocnemius muscle of CKD mice after pharmacological inhibition of Spp1).
