# Supplementary material for: Multiomics Analysis Reveals Therapeutic Targets for Chronic Kidney Disease With Sarcopenia
Source: J Cachexia Sarcopenia Muscle. 2025 Feb 6;16(1):e13696. doi: 10.1002/jcsm.13696 (PMC11799769; doi:10.1002/jcsm.13696)
Supplement: Supplementary file 1 — Table S1. Diet Composition Details. Figure S1. A representative image of Normal Control (NC) and chronic kidney disease (CKD) group. Left: NC group; Right: CKD group. Figure S2. Heatmap of the correlation analysis between the screened 29 proteins and biochemical measures and phenotypes. BUN: Blood urea nitrogen; Scr: serum creatinine; GC: gastrocnemius. *p < 0.05, **p < 0.01, ***p < 0.001. Figure S3. The effect of S100a9 recombinant protein on the myotube atrophy in vitro. (A) Western blot analysis of atrogin‐1 and murf‐1 treated with different concentrations (0, 10, 100, and 1000 ng/mL) of recombinant protein S100a9. (B) Immunofluorescence staining of myosin heavy chain (MHC) treated with different concentrations (0, 10, 100, and 1000 ng/mL) of recombinant protein S100a9. Data are expressed the mean ± SD (n = 3). Statistical significance was evaluated using a one‐way ANOVA test. Scale bars: 100 μm. *p < 0.05, **p < 0.01. Figure S4. Effect of pharmacological inhibition of Spp1 on serum creatinine and blood urea nitrogen in experimental CKD. Scr: serum creatinine; BUN: blood urea nitrogen. Table S2. Antibody list for all experiments. Table S3. Primer sequence for qPCR. [file JCSM-16-e13696-s001.docx]

**Table S1** Diet Composition Details

|  | AIN-93G | 0.2% adenine AIN-93G |
| --- | --- | --- |
| **Study Purpose** | Control diet | CKD diet |
| Dyets **Cat. #** | Dyets, 110700 | Dyets, D191101 |
| **Ingredient** | Amount (g/kg) | |
| Caisein | 200 | 200 |
| L-Cystine | 3 | 3 |
| Sucrose | 100 | 100 |
| Cornstarch | 397.486 | 397.5 |
| Dyetrose | 132 | 132 |
| Soybean Oil | 70 | 70 |
| Cellulose | 50 | 50 |
| Mineral Mix #210025 | 35 | 35 |
| Vitamin Mix #310025 | 10 | 10 |
| Choline Bitartrate | 2.5 | 2.5 |
| Adenine | 0 | 2 |
| Yellow Dye | 0 | 0.05 |

|  | AIN-93G | | 0.2% adenine AIN-93G | |
| --- | --- | --- | --- | --- |
|  | gm% | kcal% | gm% | kcal% |
| Protein | 20 | 20.3 | 20 | 20.3 |
| Carbohydrate | 64 | 63.9 | 64 | 63.9 |
| Fat | 7 | 15.8 | 7 | 15.8 |
| kcal/gm | 4 |  | 3.99 |  |

**Figure S1** A representative image of Normal Control (NC) and chronic kidney disease (CKD) group


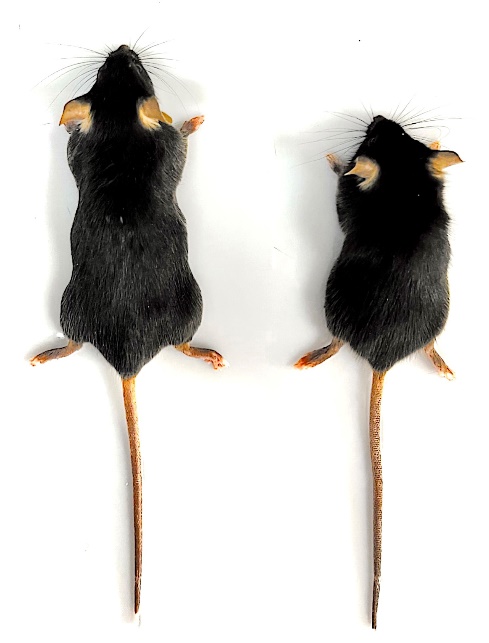


Left: NC group; Right: CKD group.

**Figure S2** Heatmap of the correlation analysis between the screened 29 proteins and biochemical measures and phenotypes


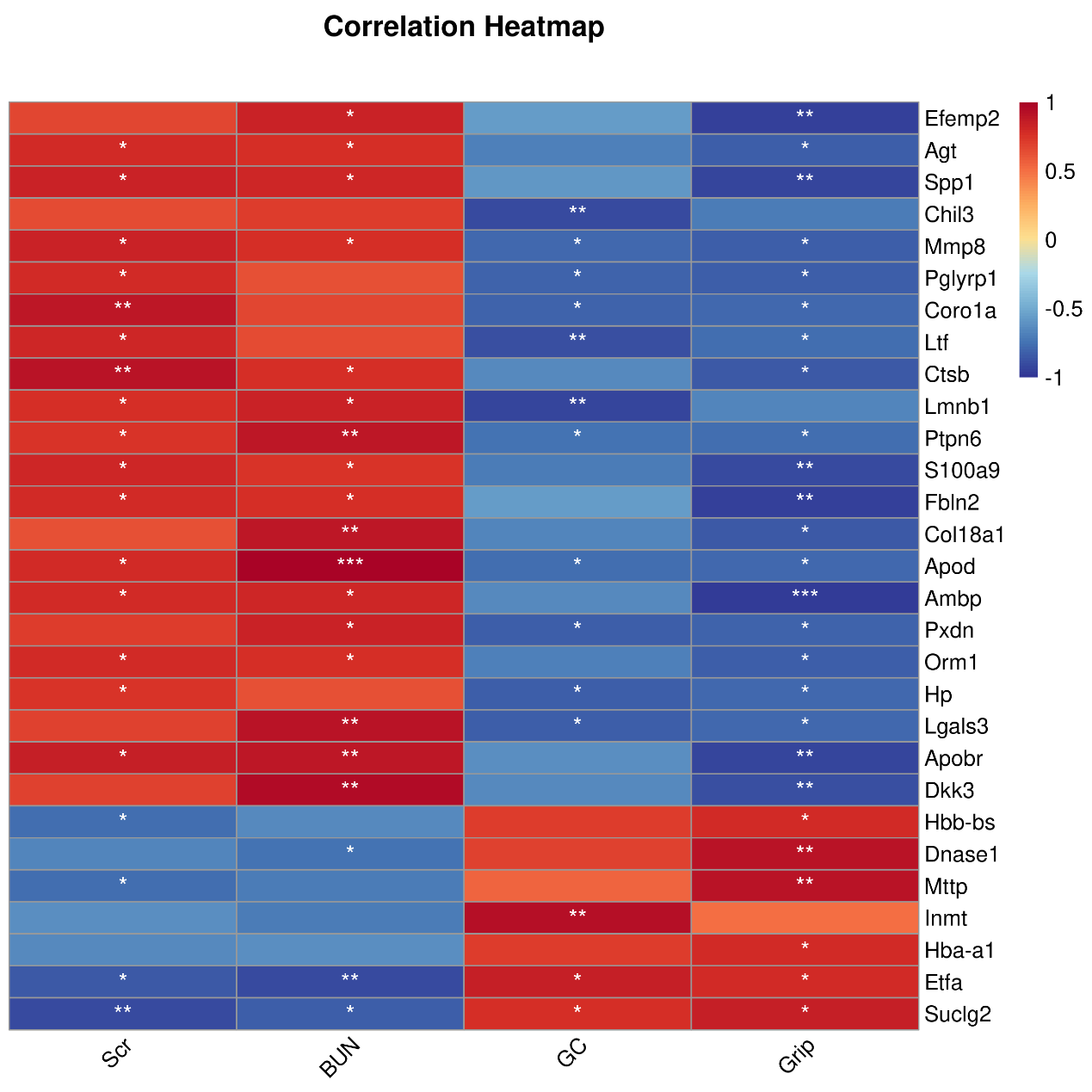


BUN: Blood urea nitrogen; Scr: serum creatinine; GC: gastrocnemius. **p* < 0.05, ***p* <0.01, ****p* < 0.001.

**Figure S3** The effect of S100a9 recombinant protein on the myotube atrophy in vitro


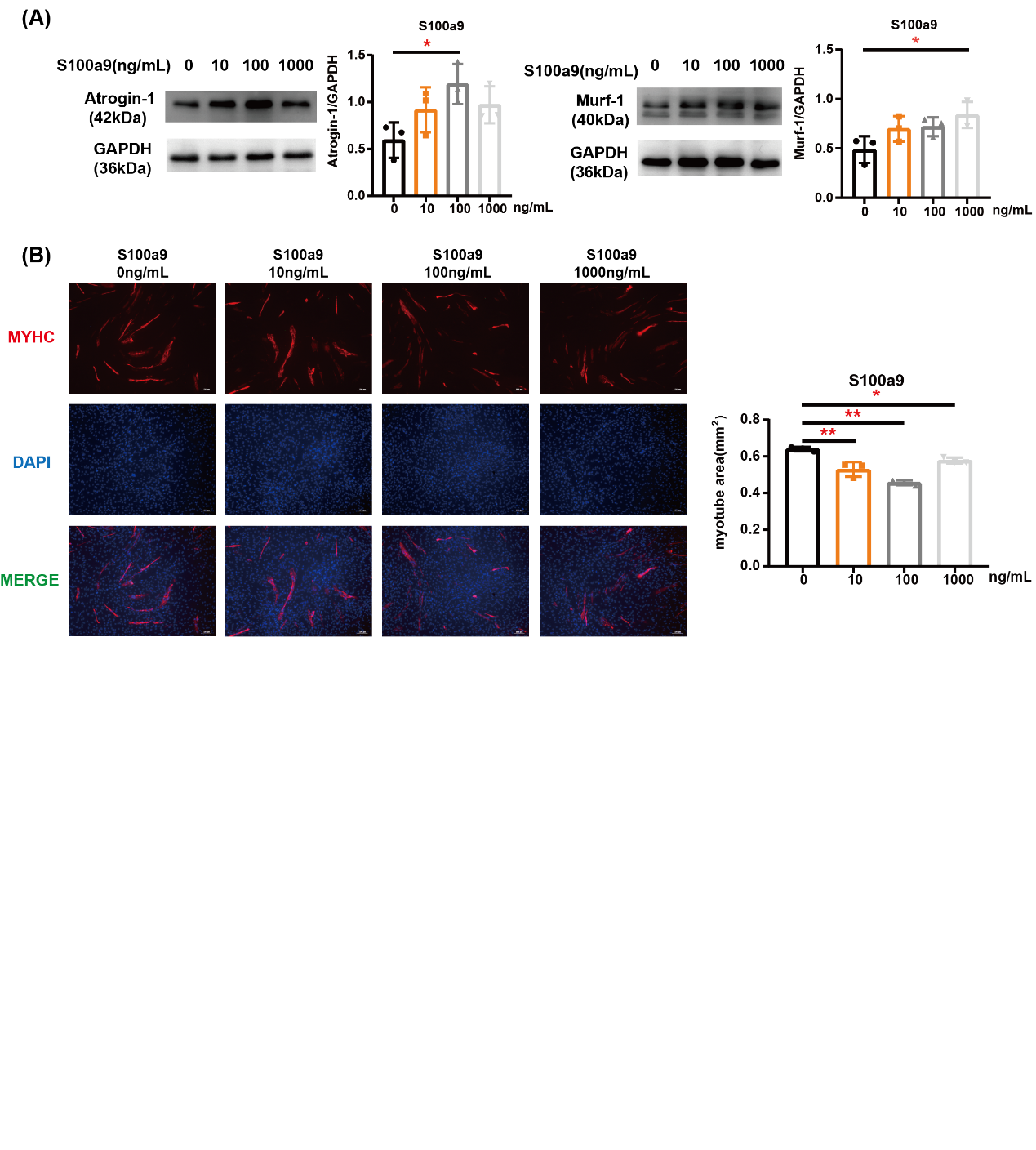


(A)Western blot analysis of atrogin-1 and murf-1 treated with different concentrations (0, 10, 100, and 1000ng/mL) of recombinant protein S100a9. (B) Immunofluorescence staining of myosin heavy chain (MHC) treated with different concentrations (0, 10, 100, and 1000ng/mL) of recombinant protein S100a9. Data are expressed the mean ± SD (n = 3). Statistical significance was evaluated using a one-way ANOVA test. Scale bars: 100μm. **p* < 0.05, ***p* < 0.01.

**Figure S4** Effect of pharmacological inhibition of Spp1 on serum creatinine and blood urea nitrogen in experimental CKD

Scr: serum creatinine; BUN: blood urea nitrogen. **p* < 0.05, ***p* < 0.01.

**Table S2** Antibody list for all experiments

| Antigen | Species | Dilution | Sipplier |
| --- | --- | --- | --- |
| Anti-Fbx32 (Atrogin-1) | Rabbit | 1:1500 | Abcam (ab168372) |
| Murf-1 | Mouse | 1:500 | Santa cruz (sc-398608) |
| Spp1 | Mouse | 1:500 for Western blotting  1:100 for immunohistochemistry | Santa cruz (sc-21742) |
| GAPDH | Rabbit | 1:5000 | Proteintech (10494-1-AP) |
| MYH | Mouse | 1:250 | Santa cruz (sc-376157) |
| Anti-rabbit IgG, HRP-linked | Goat | 1:5000 | Proteintech  (SA00001-1) |
| Anti-Mouse IgG, HRP-linked | Goat | 1:5000 | Proteintech  (SA00001-2) |
| Alexa Fluor® 594 Anti-Mouse IgG (H+L) | Donkey | 1:500 | Jackson ImmunoResearch (715-585-150) |
| Anti-Mouse IgG for immunohistochemistry | Goat | 1:200 | Servicebio (G1214-100UL) |

**Table S3 Primer sequence for qPCR**

| Gene Name | Forward Primer | Reverse Primer |
| --- | --- | --- |
| Mus musculus |  |  |
| Spp1 | AGCAAGAAACTCTTCCAAGCAA | GTGAGATTCGTCAGATTCATCCG |
| GAPDH | ACTCCACTCACGGCAAATTCA | CGCTCCTGGAAGATGGTGAT |
| Atrogin-1 | CAGCTTCGTGAGCGACCTC | GGCAGTCGAGAAGTCCAGTC |
| Murf-1 | GTGTGAGGTGCCTACTTGCTC | GCTCAGTCTTCTGTCCTTGGA |

**Materials and methods**

**RNA sequencing**

1.RNA-Seq analysis of kidneys from CKD mice was performed by the LC-Bio company (Hangzhou, China) in accordance with the manufacturer’s instructions. Total RNA (n=4 respectively for NC or CKD groups) was isolated and purified using Trizol reagent (Invitrogen, Carlsbad, CA, USA) following the manufacturer's procedure. The RNA amount and purity of each sample was quantified using NanoDrop ND-1000 (NanoDrop, Wilmington, DE, USA). The RNA integrity was assessed by Bioanalyzer 2100 (Agilent, CA, USA) with RIN number >7.0, and confirmed by electrophoresis with denaturing agarose gel. At last, we performed the 2×150bp paired-end sequencing (PE150) on an illumina Novaseq™ 6000 (LC-Bio Technology CO., Ltd., Hangzhou, China) following the vendor's recommended protocol. The mapped reads of each sample were assembled using StringTie (<http://ccb.jhu.edu/software/stringtie/>,

version:stringtie-2.1.6) with default parameters. All transcriptomes from all samples were merged to reconstruct a comprehensive transcriptome using gffcompare software ([http://ccb.jhu.edu/software/ stringtie/gffcompare.shtml](http://ccb.jhu.edu/software/%20stringtie/gffcompare.shtml), version: gffcompare-0.9.8). After the final transcriptome was generated, StringTie and ballgown (<http://www.bioconductor.org/packages/release/>bioc/html/ballgown.html) were used to estimate the expression levels of all transcripts and perform expression abundance for mRNAs by calculating FPKM (fragment per kilobase of transcript per million mapped reads) value. Genes with log2fold change>1 and adjusted *p*-value ≤0.05 were considered statistically significant.

2. RNA-Seq analysis of gastrocnemius muscle after pharmacological inhibition of Spp1 was performed by Novogene company (Beijing, China) according to the manufacturer's instructions. Total RNA was extracted from polysaccharide- and polyphenol-rich roots and leaves by using RNAprep Kit and treated with RNase-free DNase I (Qiagen). RNA quality and integrity were determined by an Agilent Bioanalyzer 2100 system (Agilent, USA) with RIN > 8.5. The RNA-Seq library was prepared by using NEBNext Ultra RNA Directional Library Prep Kit for Illumina. The cDNA library quality was quantified first by using Qubit 2.0, detected on Bioanalyzer 2100 (Agilent, USA), and then by qPCR (effective concentration > 2 nM in the library). The cDNA libraries were constructed and sequenced using the Illumina NovaSeq 6000 platform. FeatureCounts v1.5.0-p3 was used to count the reads numbers mapped to each gene. And then FPKM of each gene was calculated based on the length of the gene and reads count mapped to this gene. Genes with log2fold change>1 and adjusted *p*-value ≤0.05 were considered statistically significant.
